# Comparison of Aggressive Surgical Treatment and Palliative Treatment in Elderly Patients with Poor-Grade Intracranial Aneurysmal Subarachnoid Hemorrhage

**DOI:** 10.1155/2018/5818937

**Published:** 2018-05-28

**Authors:** Kuang Zheng, Bing Zhao, Xian-Xi Tan, Ze-Qun Li, Ye Xiong, Ming Zhong, Si-Yan Chen

**Affiliations:** ^1^Department of Neurosurgery, First Affiliated Hospital of Wenzhou Medical University, Wenzhou 325000, China; ^2^Department of Neurosurgery, Renji Hospital, Shanghai Jiaotong University School of Medicine, Shanghai 200127, China; ^3^Department of Neurology, First Affiliated Hospital of Wenzhou Medical University, Wenzhou 325000, China

## Abstract

**Objective:**

To compare the current treatment approach in elderly patients with poor-grade aneurysmal subarachnoid hemorrhage (SAH) and identify the independent predictors of the outcome after aggressive surgical treatment.

**Method:**

This prospective, multicenter cohort study included 104 poor-grade aneurysmal SAH elderly patients, 60 years or older, treated in our institution from October 2010 to March 2013. Patients were grouped according to three treatment arms. Neurological outcome was assessed using the Glasgow Outcome Scale (GOS) at baseline and at a 12-month follow-up. Univariate and multivariate analysis were performed using the following factors: sex, age, smoking history, breathing ability, alcohol consumption, cerebral hernia, aneurysm location, aneurysm diameter, WFNS grade, CT Fisher grade, treatment approach, and the timing of the aneurysm surgery.

**Results:**

At the 12-month follow-up, patients in the coiling group and clipping group had better prognosis than patients in the palliative treatment group. Univariate analysis confirmed that the treatment approach, WFNS grade, CT Fisher grade, and age are critical factors for neurological outcomes in poor-grade SAH. Multivariate analysis indicated that WFNS grade V, CT Fisher grades 3–5, and palliative treatment were independent predictors of poor prognoses.

**Conclusion:**

Aggressive surgical treatment improves the prognoses in poor-grade aneurysm elderly patients with SAH. Elderly Patients of WFNS grade IV and CT Fisher grades 1-2 are more likely to have a better outcome.

## 1. Introduction

Subarachnoid hemorrhage (SAH) refers to a clinical symptom of direct extravasation of blood into the subarachnoid space caused by ruptures of the lesional vessels located at the brain base or surface. SAH accounts for about 5% of all acute strokes and affects ~30,000 Americans each year [1]. Despite advances in diagnostic methods and surgical and perioperative treatments, the outcomes for SAH patients remain poor, with population-based mortality rates as high as 45% and severe disability rates of ~30% [2–8]. Moreover, poor-grade aneurysmal SAH patients (defined as World Federation of Neurological Surgeons (WFNS) grades IV and V) have the highest mortality and severe disability rates of all SAH types and, thus, are often treated palliatively [9]. The incidence of SAH increases with advancing age. Hence, the elderly account for a significant number of the patients presenting with SAH [10].

For poor-grade aneurysmal SAH, current therapeutic regimens include palliative treatment and aggressive surgical treatment. Palliative treatment includes the following. (1) Medical measures to prevent rebleeding: the blood pressure should be controlled to reduce the risk of rebleeding. Antiepileptic treatment should be administered in patients with clinically apparent seizures (sodium valproate). Headache is usually so severe that codeine and even a synthetic opiate might be needed. Antifibrinolytic therapy (aminocaproic acid or tranexamic acid) is administered to reduce the incidence of aneurysm rebleeding [11, 12]. (2) Prevention of cerebral vasospasm by using calcium antagonists, such as nimodipine. (3) Intracranial pressure-lowering surgery (ventricular drainage surgery or lumbar drainage, hematoma evacuation, and decompressive craniectomy). Aggressive surgical treatment includes (1) Neurosurgical Clipping treatment of ruptured intracranial aneurysm combined with or without intracranial pressure-lowering surgery and (2) endovascular coiling treatment of ruptured intracranial aneurysm combined with or without intracranial pressure-lowering surgery.

Most of these poor-grade elderly aneurysms patients had been treated with palliative measures in the past, thereby delaying life-saving neurosurgical clipping or endovascular treatment, but this approach was usually associated with a poor outcome, over 90% of SAH patients die during palliative treatment [13]. In a few researches, surgery was contraindicated in those elderly patients with a poor clinical grade and suggested that those with worse grades do poorly with or without aggressive intervention [14, 15]. Some authors have recommended early aneurysm surgery, including clipping or coiling, and active medical treatment in elderly patients; however, most groups have reported poor outcomes in patients with poorer clinical grades and aggressive surgery is not recommended in elderly patients with a poor clinical grade [16, 17]. On the other hand, Lan et al. reported that in a group of elderly patients with poor-grade aneurysms of clinical treatment and follow-up data they found 40% of patients with good prognosis, and mortality is 45% [18]; the result is encouraging. So far, the attitude towards such patients is still controversial. Therefore, it is particularly important to carry out a multicenter clinical study to investigate the prognosis of poor-grade elderly patients with aneurysms and potential prognostic factors.

We conducted a multicenter, prospective, cohort study on 104 poor-grade aneurysm elderly patients between 2010 and 2013. The primary purpose of this study was to evaluate whether different treatment approaches (palliative treatment, clipping, or coiling) for poor-grade aneurysmal SAH affect elderly patient prognosis. Functional outcome was assessed by the Glasgow Outcome Scale (GOS). Other potential factors, including sex, age, smoking history, breathing ability, alcohol consumption, cerebral hernia, aneurysm location, aneurysm diameter, WFNS grade, CT Fisher grade, and the timing of aneurysm surgical treatment, were also collected and analyzed. We hypothesized that the treatment approach is a critical factor for neurological outcomes in poor-grade aneurysmal SAH elderly patients.

## 2. Materials and Methods

### 2.1. Study Design and Patient Population

This was a multicenter, prospective clinical study conducted between October 2011 and October 2014, involving 11 clinic medical centers. The clinical registration number is ChiCTR-OCH-10001041 and the approval number from the Ethics Committee is ChiECRCT-2010019. A total of 104 patients were enrolled from October 2010 to March 2013. The inclusion criteria were as follows: (1) being older than 60 years of age; (2) WFNS grade of IV to V during hospitalization or any preoperative period; (3) diagnosis of spontaneous SAH, confirmed radiologically or by lumbar puncture; and (4) diagnosis of intracranial aneurysm confirmed surgically or by the digital subtraction angiography (DSA), computed tomographic angiography (CTA), and magnetic resonance angiography (MRA) examinations, and the SAH was directly caused by the confirmed aneurysms. The exclusion criteria were as follows: (1) patients suffering from an aneurysmal SAH at a WFNS grade of III or less during the hospital stay and preoperatively; (2) uncertain diagnosis of intracranial aneurysm, or the responsible aneurysm being surgically treated in a noncentered hospital; (3) diagnosis of an identified intracranial aneurysm that is not responsible for the SAH; (4) intracranial hematoma complications that are not relevant to the responsible intracranial aneurysm; and (5) expected survival of less than 1 year due to severe diseases of the other systems, such as Carcinoma of pancreas. All the authorized relatives of patients signed informed consent forms and agreed to cooperate with our clinical treatments and follow-ups.

### 2.2. Patient Characteristics

We recorded sex, age, smoking history, breathing ability, alcohol consumption, cerebral hernia, aneurysm location, aneurysm diameter, WFNS grade, CT Fisher grade, treatment approach, and the timing of the aneurysm surgery as potential prognostic predictors. We evaluated classified aneurysms based on size as follows: <3 mm as a small-size aneurysm, 3–10 mm as a middle-size aneurysm, and >10 mm as a large-size aneurysm. Cerebral computed tomography (CT) was performed for all the patients immediately after their admission, and CT reexaminations were applied according to the dynamics in medical conditions. The cerebral CT Fisher scale was adopted to classify the SAH based on severity. According to age, the patients were divided into two groups; the age of 70 years old was set as the dividing line. The timing of the aneurysm surgery was divided as follows: (A) ultra-early treatment defined as aneurysm treatment within 24 h after intracranial aneurysm rupture and hemorrhage; (B) early treatment defined as aneurysm treatment between 24 h and 72 h after intracranial aneurysm rupture and hemorrhage; and (C) mid–late treatment defined as aneurysm treatment more than 3 d after intracranial aneurysm rupture and hemorrhage.

### 2.3. Treatment Groups

In this study, patients were divided into three groups based on their treatment. The palliative treatment group included patients who merely received either conservative drug treatment or simple intracranial pressure-lowering surgery. The clipping group included patients who underwent neurosurgical clipping surgery or neurosurgical clipping combined with intracranial pressure-lowering surgery. Finally, the coiling group included patients who underwent endovascular coiling or coiling combined with intracranial pressure-lowering surgery.

### 2.4. Patient Outcomes and Follow-Up

Neurological function testing was performed as the outcome measure at 12 months postoperatively, either in our outpatient clinic or by the telephone. Neurological function was evaluated by the Glasgow Outcome Scale (GOS) as follows: score 5 indicated that patient had light damage with minor neurological and psychological deficits; score 4 indicated that the patient had no need for assistance in everyday life, and employment was possible but they may require special equipment; score 3 indicated that the patient had severe injury with a permanent need for help with daily living; score 2 indicated that the patient was in a persistent vegetative state with only minimal responsiveness (e.g., patients' eyes open with the sleep-wake cycle); and score 1 indicated death.

Many references classify the GOS scores 4 and 5 as “good prognoses” and scores 3, 2, and 1 as “poor prognoses” [19]. In the present study, we classified patients with a GOS score of 4-5 points as “good prognoses” (i.e., well restored or only mildly disabled), those with a GOS score of 2-3 points as “poor prognoses” (i.e., severely disabled or in a vegetative state), and those with a score of 1 point as “dead”.

### 2.5. Statistical Methods

Data were expressed as frequency (percentage), and all statistical analyses were performed using the IBM SPSS Statistic v22.0 and SAS v9.4 software packages. Univariate analysis was performed to assess the other potential prognostic predictors for SAH. Comparison between two groups was analyzed using the Wilcoxon rank-sum test; comparison among multiple groups was analyzed by the Kruskal-Wallis *H* test, and multiple comparisons were analyzed by the Nemenyi test, with *P* < 0.05 indicating statistical significance. In the multivariate analysis, the multilevel logistic regression was utilized using the GOS scores at 12 months postoperatively as the dependent variable and factors from the univariate analysis with a *P* < 0.1 as independent variables, with *P* < 0.05 indicating statistical significance.

## 3. Results

### 3.1. Baseline Characteristics of the Patients

**Figure 1:**
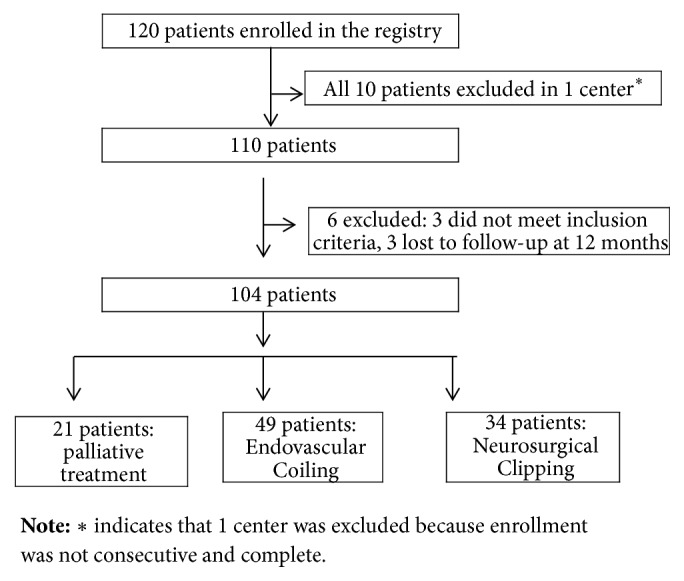
Study flow diagram.

A total of 104 patients (40 males and 64 females) were enrolled in the study (Figure 1). The baseline characteristics for all patients are shown in Table 1. Briefly, there were 51 WFNS grade IV patients and 53 WFNS grade V patients, 17 cerebral CT Fisher grade 1-2 patients, and 87 cerebral CT Fisher grade 3–5 patients. There were 3 patients with an intraoperatively confirmed aneurysm, and the remaining 101 patients underwent CTA or DSA examinations to confirm their diagnoses. There were 94 cases of intracranial anterior circulation aneurysms and 10 cases of posterior circulation aneurysms. According to the maximal aneurysm diameters, there were 13 small-size aneurysms, 84 middle-size aneurysms, and 7 large-size aneurysms.

### 3.2. Treatments for SAH

There were 49 patients treated with aneurysm coiling: single coiling was performed for 34 patients and coiling in combination with intracranial pressure-lowering surgery for 15 patients. There were 34 patients treated with aneurysm clipping: single neurosurgical clipping for 10 patients and neurosurgical clipping in combination with intracranial pressure-lowering surgery for 24. 21 patients received palliative treatment: single external ventricular drainage for 2 patients and merely conservative drug treatment for 19 patients. Of the 83 patients undergoing aneurysm treatments, 32 accepted the ultra-early surgeries, 29 received early surgeries, and 22 took mid–late surgeries.

### 3.3. Patient Outcomes at 12-Month Follow-Up

After 12 months, 23 (46.9%) patients in the coiling group had died, 8 (16.3%) were with a poor prognosis, and 18 (36.7%) were with a good prognosis, while, in the clipping group, 11 (32.4%) died, 10 (29.4%) were with a poor prognosis, and 13 (38.2%) were with a good prognosis at the 12-month follow-up. For the 21 patients in the palliative treatment group, 12 patients died during hospitalization, 5 patients died after 1 month, 2 suffered from poor prognosis, and 4 patients achieved good prognosis at the postoperative 12-month follow-up. The outcome in palliative treatment group with coiling group was significantly different (*P* = 0.024). The outcome in palliative treatment group with clipping group was significantly different (*P* = 0.002); however, no significant difference was observed between the coiling and clipping groups (*P* = 0.267) (Table 2).

### 3.4. Identification of Potential Prognostic Factors

Univariate analysis used the GOS scores at the 12-month follow-up as the dependent variable. Analysis result (Table 3) indicated that the prognoses in WFNS grade IV patients were significantly better than WFNS grade V patients; prognoses in patients with a low CT Fisher grade (grades 1 and 2) were significantly better than those with a high grade (grades 3, 4, and 5); prognoses in younger patients (60–70 years old) were significantly better than those older patients (≥70 years old); prognoses in patients from the coiling group and clipping group were significantly better than those from the palliative treatment group.

### 3.5. Multivariate Analysis of Prognostic Factors for Aneurysmal SAH

We conducted a multivariate analysis by adopting the GOS scores at the 12-month follow-up as the dependent variables and factors from our univariate analysis with a *P* < 0.1 as the independent variable (Table 4). After controlling for the confounders, WFNS grade, CT Fisher grades, and therapeutic approach were identified as significant prognostic predictors (*P* < 0.05). As for the therapeutic approach, the coiling group had an odds ratio (OR) value of 13.777 (*P* < 0.05), while the clipping group had an OR value of 7.807 (*P* < 0.05) compared to palliative treatment group, indicating that prognoses in patients from the coiling group and clipping group were better than using a palliative approach. As for the CT Fisher grade, compared to patients with grades 3–5, grade 1-2 patients had an OR value of 3.111 (*P* = 0.025), indicating that prognoses in CT grade 1-2 patients were better than for grade 3–5 patients. As for the admission grade (WFNS), compared to the grade V patients, grade IV patients had an OR value of 4.482 (*P* < 0.05), indicating that prognoses in admission grade IV patients were better than grade V patients.

## 4. Discussion

Aneurysmal SAH elderly patients often suffer higher rate of rebleeding and hydrocephalus, more serious cerebral vasospasm [18, 20–22]. The proportion of poor-grade aneurysms in the elderly group was significantly higher than the younger group; this part of the patients are often facing a huge therapeutic challenge. Another significant feature of aneurysmal SAH elderly patient is often preexisting health problems such as hypertension, diabetes, chronic bronchitis, atherosclerosis, coronary heart disease, emphysema and cardiopulmonary, and renal dysfunction and other systemic diseases. Subarachnoid hemorrhage is likely to further exacerbate these problems, making it difficult to tolerate surgery in some cases. Considering the significant cerebral edema, high intracranial pressure, surgical operation difficulties, more complications, and poor prognosis, the majority of medical centers choose palliative treatment for elderly poor-grade aneurysm patients.

While developments in neurosurgery and imaging technologies have improved aneurysm treatment success, the mortality and disability rates of poor-grade aneurysmal SAH elderly patients remain high. Here we evaluated the effects of different treatment methods, both palliative treatment and aggressive treatment (including clipping and coiling) on neurological outcomes in poor-grade SAH elderly patients. Our univariate analysis confirmed that the treatment approach is a critical factor for neurological outcomes in poor-grade SAH. We found that active surgical treatment for aneurysm improves the prognoses in poor-grade aneurysmal SAH elderly patients. Moreover, our multivariate analysis showed that WFNS grade V patients, CT Fisher grades 3–5 patients, and those who adopted a palliative treatment had poor functional outcomes.

Previous studies have indicated that conducting aneurysm surgical intervention during the ultra-early and early periods of aneurysmal SAH is not recommended, possibly due to unstable vital signs and high intracranial pressure. Therefore, aggressive surgical interventions are generally only given according to specific conditions observed after the conservative drug treatment and intensive care. However, early rerupture of the aneurysm usually occurs within 24 h after the first bleeding, with risks as high as 87% [23]. Moreover, the risk of early rerupture of the aneurysm in poor-grade aneurysmal SAH (Hunt-Hess grade III-IV) is significantly higher [24, 25]. Therefore, early surgical intervention may be beneficial in preventing ultra-early rebleeding, as reported previously [26].

The effectiveness of neurosurgical clipping compared to endovascular coiling in poor-grade SAH is still debated. For neurosurgical clipping, a prognostic study by Bailes et al. [13] showed that the mortality rate and improvement rate of poor-grade aneurysm patients were 50% and 35%, respectively. Similarly, Le Roux et al. [27] found that mortality rate and improvement rate of poor-grade aneurysm patients undergoing neurosurgical clipping were 43% and 38%, respectively. On the other hand, as a novel minimally invasive surgical approach, endovascular coiling has been increasingly applied internationally. Many large-scale studies have compared the therapeutic efficacy of coiling and clipping, including the International Subarachnoid Aneurysm Trial (ISAT) [28]. The study reported that the 1-year disability-free survival in the coiling group was higher than the clipping group [28]. However, the 5-year ISAT follow-up results showed that although coiling still had an advantageous in mortality over clipping, the benefit was no longer significant [29]. Although the ISAT studies are very famous in research of clipping and coiling, there are biases in their patient selections: low-grade (grades I and II) aneurysms account for more than 88% of the subjects, the average age is 54 years old; therefore, their data may be unreliable for the treatment of poor-grade (grades IV and V) aneurysm elderly patients. In this study, the analyses of grades IV and V aneurysm patients showed no statistical difference in efficacy between the clipping and coiling groups, which was inconsistent with some of the previous studies. A potential reason for the inconsistencies between our results and others is the selection of patients. In the present study, the research subjects were elderly poor-grade aneurysmal SAH patients, while subjects in the previous studies were mainly younger, low-grade aneurysm patients or those with unruptured aneurysms. Since the patients' preoperative conditions in the previous studies were generally better, the factors associated with prognoses might vary, thereby explaining the different results.

The WFNS grading system can be used to observe the changes in disease conditions of patients with spontaneous SAH and predict prognoses, thereby facilitating patient management [19]. In two studies conducted by Mocco et al. [30] and Chiang et al. [31], significant differences were observed in the SAH prognoses based on WFNS grade (i.e., WFNS grade V patients had 7% and 21% good prognosis rates, WFNS grade IV patients had 38% and 62% good prognosis rates in the two studies, respectively). In a recent single-center study on 248 patients with poor-grade SAH (WFNS grade IV or V), favourable outcomes were significantly associated with lower initial WFNS grades (*P* < 0.0001) [32]. Similarly, our study also indicated that WFNS grade was a predictor in the prognosis of aneurysmal SAH.

Delayed neurological dysfunction caused by vasospasm is one of the main reasons leading to the poor prognosis in poor-grade aneurysmal SAH patients. Vasospasm is closely correlated with the CT Fisher grade in SAH patients, increased SAH results stimulate the release of vasoconstrictors, leading to delayed vasospasm [33]. Most patients with a high CT Fisher grade (grades 3, 4, and 5) are reported to suffer from delayed vasospasm [34]. Our study also showed that prognoses in patients with different CT Fisher grade were significantly different. Therefore, prompt pressure-raising, volume-dilating, and external ventricular drainage, or lumbar puncture induced subarachnoid catheter drainage of bloody cerebrospinal fluid and other measures, should be performed after the early treatment to improve cerebrovascular vasospasm in poor-grade aneurysmal SAH.

There were some limitations in our study. Firstly, the disadvantages associated with the prospective cohort study design, not a randomized one. Besides, potential risk factors like family income and hospitalization cost, which may also affect treatment decision-making, were not collected and analyzed in our study. This study is multicentric, intensive care unit management was not highly standardized, the ICU stay was not described in this cohort, and survival may be dependent on intensive care unit management, such as incidence of pneumonia and ICU length of stay. In particular, we only compared outcomes after 12 months in this study. Therefore, a longer follow-up period is required to further verify our results. In future prospective and multicenter study featuring different intensive care unit therapeutic strategies should be conducted to analyze the long-term prognosis of elderly patients with poor-grade intracranial aneurysmal subarachnoid hemorrhage after different treatments.

In summary, active surgical treatment for aneurysms improves the prognoses in poor-grade aneurysmal SAH elderly patients compared with those who adopt a palliative treatment. In future, rapid surgical intervention for poor-grade aneurysmal elderly patients should be provided in order to avoid the catastrophic outcomes of hemorrhage and improve elderly patient mortality and disability rates.

## Figures and Tables

**Table 1:** Baseline characteristics of the study group.

Characteristic	group	Total	palliative	Coiling	Clipping	*P* value
Gender	Male Female	40 64	11 10	18 31	11 23	0.314

Age	60–70 years old ≥70 years old	75 29	16 5	35 14	24 10	0.565

Alcohol consumption	Never Mild Moderate to severe	77 14 13	16 2 3	34 9 6	27 3 4	0.734

Smoking	Never Yes	71 33	13 8	30 19	28 6	0.099

Breathing status	Unsteady Steady	9 95	1 20	4 45	4 30	0.659

Cerebral hernia	No Yes	89 15	18 3	44 5	27 7	0.146

WFNS Grade	IV V	53 51	9 12	26 23	18 16	0.708

Head CT Fisher Grade	1-2 3–5	17 87	6 15	6 43	5 29	0.227

Aneurysm location	Anterior circulation Posterior circulation	94 10	19 2	42 7	33 1	0.226

Aneurysm diameter	≤3 mm 3–10 mm ≥10 mm	13 84 7	3 17 1	8 39 2	2 28 4	0.441

The timing of the aneurysm surgery	Ultra-early (<24 h) Early (24–72 h) Mid–Late (>3 d)	32 29 22	0 0 0	18 20 11	14 9 11	0.363

**Table 2:** Summary of SAH patient outcomes after 12 months in the three treatment groups.

Treatment group	Died	Poor Prognoses	Good Prognoses	*P* value^*∗*^
palliative	17 (81.0%)	2 (9.5%)	2 (9.5%)	0.024
Coiling	23 (46.9%)	8 (16.3%)	18 (36.7%)	0.264
Clipping	11 (32.4%)	10 (29.4%)	13 (38.2%)	0.002

*Note*. ^*∗*^Fisher's Exact Test; *P* value (palliative group versus coiling group) = 0.024; *P* value (clipping group versus coiling group) = 0.264; *P* value (palliative group versus clipping group) = 0.002.

**Table 3:** Univariate analysis of the potential prognostic factors.

Factor	Group	Sample size	Mean rank	OR value	Confidential interval of the OR value	*P* value
Sex	Male	40	49.48	0.699	0.329–1.484	0.381
	Female	64	54.39			

Age	60–70 years old	74	56.03	1.845	1.170–2.910	0.046
	≥70 years old	30	45.13			

Smoking	Never	72	53.45	1.261	0.571–2.787	0.564
	Yes	32	50.36			

Alcohol consumption	Never	78	52.47	0.992	0.430–2.293	0.932
	Yes	26	52.58			

Aneurysm diameter	≤3 mm	14	51.32	1.062	0.128–8.820	0.825
	3–10 mm	86	52.79	1.174	0.174–7.933	
	≥10 mm	4	50.38	1.000		

Breathing status	Unsteady	11	42.14	0.501	0.142–1.765	0.191
	Steady	93	53.73			

Cerebral hernia	No	95	54.26	4.730	0.892–25.103	0.093
	Yes	9	33.89			

Aneurysm location	Anterior circulation	93	52.11	0.799	0.248–2.573	0.453
	Posterior circulation	11	55.82			

WFNS grade	IV	53	63.44	5.048	2.307–11.045	<0.001
	V	51	41.13			

Head CT Fisher grade	1 and 2	24	62.92	2.573	1.080–6.129	0.008
	3, 4 and 5	80	49.38			

treatment approach	clipping	34	60.15	7.360	2.092–25.868	0.004
	coiling	49	54.57	5.233	1.567–17.479	0.007
	palliative	21	35.29	1.000		

The timing of the aneurysm surgery	Ultra-early surgery	32	38.13	0.839	0.310–2.277	0.732
	Early surgery	29	50.64	2.450	0.878–6.835	0.087
	Mid–late surgery	24	40.27	1.000		

**Table 4:** Multivariate analysis of the potential prognostic factors.

Variable	Regression coefficient	OR	Confidential interval of the OR value	*P* value
treatment approach				
Coiling (versus palliative)	2.623	13.777	3.380–56.149	<0.001
Clipping (versus palliative)	2.055	7.807	2.081–29.283	0.002
CT Fisher grade (low versus high)	1.135	3.111	1.147–8.449	0.026
WFNF grade (IV versus V)	1.500	4.482	1.912–10.496	0.001
Cerebral hernia (symmetric versus asymmetric)	1.551	4.716	0.745–29.904	0.100
Age (60–70 years old versus ≥70 years old)	0.468	1.597	0.935–2.729	0.087

## Data Availability

The data generated and analyzed during the current study are not publicly available since the research database relates to a clinical case study, including patient's name and other private data. They are, however, available from the corresponding author on reasonable request.
